# Polyimide-Based Membrane Materials for CO_2_ Separation: A Comparison of Segmented and Aromatic (Co)polyimides

**DOI:** 10.3390/membranes11040274

**Published:** 2021-04-08

**Authors:** Andrzej Jankowski, Eugenia Grabiec, Klaudia Nocoń-Szmajda, Andrzej Marcinkowski, Henryk Janeczek, Aleksandra Wolińska-Grabczyk

**Affiliations:** Centre of Polymer and Carbon Materials, Polish Academy of Sciences, 34 M. Curie-Sklodowska Str., 41-819 Zabrze, Poland; ajankowski@cmpw-pan.edu.pl (A.J.); egrabiec@cmpw-pan.edu.pl (E.G.); knocon@cmpw-pan.edu.pl (K.N.-S.); amarcinkowski@cmpw-pan.edu.pl (A.M.); hjaneczek@cmpw-pan.edu.pl (H.J.)

**Keywords:** CO_2_ selective membrane, segmented copolymer, poly(ethylene oxide), polyimide

## Abstract

A series of new poly(ethylene oxide) (PEO)-based copolyimides varying in hard segment structure are reported in this work as CO_2_ selective separation membranes. Their structural diversity was achieved by using different aromatic dianhydrides (4,4′-oxydiphthalic anhydride (ODPA), 4,4’-(hexafluoroisopropylidene)diphthalic anhydride (6FDA)) and diamines (4,4′-oxydianiline (ODA), 4,4′-(4,4′-isopropylidene-diphenyl-1,1′- diyldioxy)dianiline (IPrDA), 2,3,5,6-tetramethyl-1,4-phenylenediamine (4MPD)), while keeping the content of PEO (2000 g/mol) constant (around 50%). To get a better insight into the effects of hard segment structure on gas transport properties, a series of aromatic polyimides with the same chemistry was also studied. Both series of polymers were characterized by ^1^HNMR, FTIR, WAXD, DSC, TGA, and AFM. Permeabilities for pure He, O_2_, N_2_, and CO_2_ were determined at 6 bar and at 30 °C, and for CO_2_ for pressures ranging from 1 to 10 bar. The results show that OPDA-ODA-PEO is the most permeable copolyimide, with CO_2_ permeability of 52 Barrer and CO_2_/N_2_ selectivity of 63, in contrast to its fully aromatic analogue, which was the least permeable among polyimides. 6FDA-4MPD-PEO ranks second, with a two times lower CO_2_ permeability and slightly lower selectivity, although 6FDA-4MPD was over 900 times more permeable than OPDA-ODA. As an explanation, partial filling of hard domain free voids by PEO segments and imperfect phase separation were proposed.

## 1. Introduction

Control of CO_2_ emissions is one of the most challenging issues the world is facing today. To cope with this problem, actions in many fields must be undertaken, especially in areas improving energy efficiency, using renewable energy resources, and reducing the post-combustion CO_2_ emissions. Various separation techniques have been developed to capture CO_2_ at power plants, including solvent absorption, adsorption, cryogenic distillation, and membrane separation [1,2,3]. Among them, the application of membranes as alternative, environmentally friendly technology appears to have gained much interest in recent years. When compared with other technologies, the main advantages of membrane gas separation are its simplicity, compactness, flexibility in operation and integration with already existing systems, low capital costs, as well as lower energy consumption. It also does not require the addition of potentially expensive and/or difficult to handle chemicals.

The progress in membrane post combustion CO_2_ capture has been widely reviewed from different perspectives, including the developments in membrane material design, process engineering, and engineering economics [4,5,6,7,8,9,10].

Current gas separation membrane technologies are dominated by dense solution diffusion polymeric membranes. This is due to the attractive combination of low costs with facile processing and innovation that characterizes polymer materials, as well as the good mechanical stability, high films and hollow fibers forming capability, and the simple scalability of such membranes. For practical reasons, polymeric membranes can be divided into two groups: rubbery and glassy. The difference between them is determined by the temperature of polymer glass transition (*T*_g_), i.e., rubbery polymeric membranes operate above their *T*_g_, while glassy ones operate below their *T*_g_. The gas permeability through polymers from both groups can differ by as much as several orders of magnitude, and there is a general rule that rubbery membranes are more permeable than the glassy ones, whereas the reverse trend is observed with respect to membrane overall selectivity. This trade off behavior has been demonstrated by Robeson, who expressed it in the form of an upper bound relationship between the log of the selectivity versus the log of the permeability for a faster permeating gas [11,12]. Overcoming the upper bound relationship is the focus of intense research carried out in the area of gas separation polymeric membranes. For the application to CO_2_ post combustion capture, membrane performance can be improved either by increasing the solubility or diffusivity of CO_2_ in the membrane. The first approach can be accomplished by introducing polar groups able to interact with CO_2_ into a polymer, thereby increasing its solubility relative to nitrogen. The second can be achieved by obstructing polymer chain packing while at the same time hindering backbone mobility that favors the diffusion of smaller CO_2_ molecules. The various combinations of these approaches have produced a wide range of polymeric membranes showing high potential for carbon capture.

One of the interesting groups of materials is segmented (multiblock) copolymers composed of alternating flexible and rigid segments. Due to their incompatibility, the segments undergo microphase separation resulting in a two-phase system, where glassy or semi-crystalline hard segment domains serve as virtual crosslinks and reinforcing filler for the rubbery matrix. By varying the molecular structure, their length and composition, of the segments, the properties of the material can be substantially modified. Among the segmented copolymers, those having soft segments based on poly(ethylene oxide) (PEO) aroused particular interest in terms of their application for CO_2_ post combustion capture [10,13,14]. In addition to commercially available PolyActive^®^ (OctoPlus N.V., Leiden, The Netherlands), and Pebax^®^ (Arkema, Colombes, France) materials, a number of other poly(ether ester) [15], poly(ether amide) [16], and poly(ether imide) [17,18,19,20,21,22,23,24,25,26,27,28] copolymers have been synthesized and their gas transport properties have been reported. As indicated by the results of these studies, the PEO-based copolymers exhibit high CO_2_ permeability and high CO_2_/N_2_ selectivity. It has also been noted that membrane gas selectivity is less influenced by the copolymer structure variation than the permeability, which was found to depend strongly on the copolymer morphology. In this group of membrane materials, poly(ether imide)s have attracted particular attention owing to the expected benefits associated with the presence of polyimide units, as aromatic polyimides show very good gas transport properties and a number of other outstanding properties, such as excellent thermal stability, chemical resistance, film forming ability, and mechanical durability [29]. Since it has been anticipated that the PEO phase creates a major pathway for gas permeation, the research directions have been focused primarily on studying the impact of the PEO segment length and content on the material properties. For example, Tena et al. [23] and Munoz et al. [20] have investigated copolymers based on 3,3’,4,4’-biphenyltetracarboxylic dianhydride (BPDA), 4,4′-oxydianiline (ODA), and PEO having a molecular weight of 2000 and 6000 g/mole, respectively. Varying the percentages of PEO from 10 to 68 wt.%, the authors found that gas permeability increases nonlinearly with the increasing amount of PEO-2000 and shows a maximum at 43 wt.% for the PEO-6000-based copolymers. In both cases, an enhancement in permeation with thermal treatment was observed, which was associated with improved phase separation. The increase in permeability with increasing PEO 1900 content has also been reported for the copolymers based on pyromellitic dianhydride (PMDA) and ODA [19]. However, for copolymers synthesized from PEO 2000, pentiptycene-based diamine and 6FDA dianhydride, a different permeation behavior has been reported [27]. The minimum in the permeability curve at 40 wt.% PEO content was noted, and this was attributed to the transition of these copolymers from being diffusivity-selective to solubility-selective. On the other hand, for a given PEO weight percentage, gas permeabilities were found to increase with an increasing PEO molar mass up to 2000 g/mol [21,27], and to become constant for the PEOs with higher molar masses, provided there is no crystallinity in the PEO-based domains, e.g., when the permeability measurements are performed at a temperature above PEO crystal melting [21,24]. Otherwise, a maximum in the permeability curve has been observed, which was attributed to the balance of the opposite effects of PEO segment crystallization, slowing down gas permeation, and the improvement of phase separation raising the permeation rate [21]. Relative to several papers on the correlations between PEO segment length and content and membrane gas transport properties, research studies on the impact of the hard segment chemical structure on the gas transport are rather limited. Tena et al. [22] studied copolymers based on BPDA, PEO 2000, and different aromatic diamines. They found that the incorporation of an aromatic diamine with a flexible ether linkage (ODA) into a hard segment, in place of a shorter or more rigid one, caused a reduction in permeability and a slight improvement in selectivity as a result of the less perfect phase separation. On the other hand, Chen et al. [19] reported that the gas separation performance of the PEO 2000, and ODA diamine-based copolymers was significantly affected by the variations in the kind of dianhydride. They noticed the highest permeability for the PMDA-based copolymer, and the lowest for the 4,4’-(hexafluoroisopropylidene)diphthalic anhydride (6FDA)-based one. Again, the permeability differences were explained by the differences in the degree of phase separation among copolymers, with the 6FDA-based copolymer showing a tendency for hard and soft segment mixing.

**Scheme 1:**
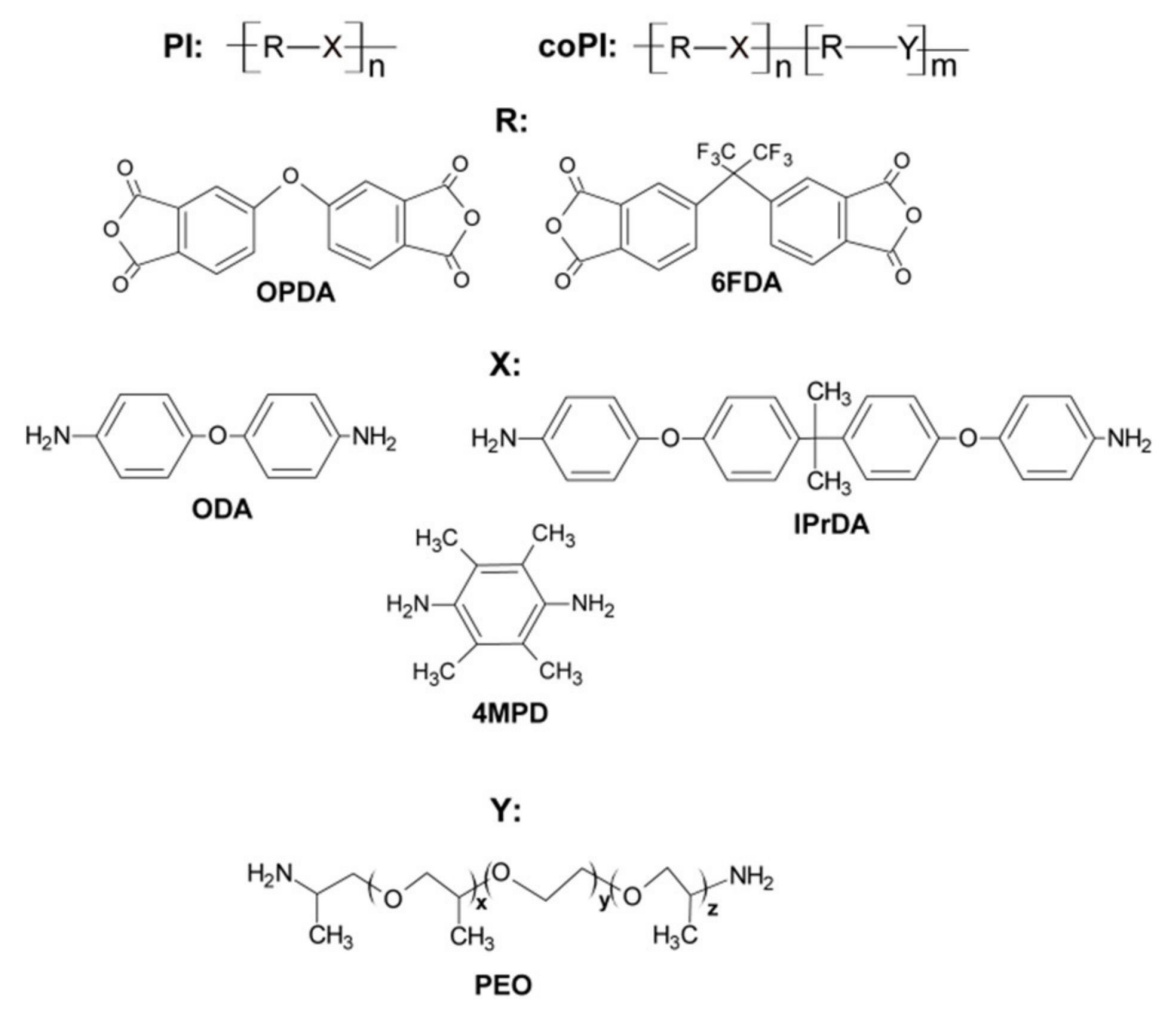
Chemical structures of the aromatic (PI) and segmented (co)polyimides (coPI).

The aim of this paper was to extend the current understanding of gas separation with PEO-based segmented copolymers by investigating several copolyimides with varying hard segment chemical structures. To get a better insight into the effects of hard segment structural changes on gas transport properties, a series of pure aromatic polyimides with the same chemistry were also studied. These polyimides were obtained from two different dianhydrides and three different aromatic diamines, the structure of which is depicted in Scheme 1. The four PEO-based copolyimides were prepared from the presented aromatic diamines and dianhydrides, and PEO with a molar mass of 2000 g/mol, using an aromatic diamine/PEO molar ratio of 3:1. The aromatic monomers were selected in order to obtain polyimides presenting large differences in gas transport properties. This was expected to result in variations in physical properties and the gas permeation behavior of the PEO-based copolymers as well, enabling a comprehensive study on structure–property relationships. To the best of our knowledge, these copolymers have not been studied for gas separation yet.

## 2. Materials and Methods

### 2.1. Materials

4,4′-Oxydiphthalic anhydride (OPDA), 4,4′-(4,4′-isopropylidene-diphenyl-1,1′- diyldioxy)dianiline (IPrDA), and 4,4′-oxydianiline (ODA) were purchased from Sigma-Aldrich S.A. 4,4’-(Hexafluoroisopropylidene)diphthalic anhydride (6FDA), and 2,3,5,6-tetramethyl-1,4-phenylenediamine (4MPD) were obtained from Tokyo Chemical Industry Co., Ltd. (Tokyo, Japan). Bis(2-aminopropyl)poly(ethylene oxide) (PEO), Jeffamine ED-2003 with a molar mass of 2000 g/mol, was kindly donated by Huntsman (Rotterdam, The Netherlands). Anhydrous N-methyl-2-pyrrolidinone (NMP), and 1,2-dichlorobenzene (ODB) were received from Sigma-Aldrich Poland S.A. (Poznań, Poland). N,N-Dimethylformamide (DMF) and methanol were purchased from Avantor Performance Materials Poland S.A. (Gliwice, Poland)

Nitrogen, carbon dioxide, helium with a purity of 99.998% and oxygen (99.95%) were supplied from Messer Poland S.A. (Chorzów, Poland), and they were used as received.

4,4′-Oxydianiline was recrystallized from methanol in the presence of decolorizing charcoal. DMF was vacuum distilled before use. The other reagents were used without further purification. 6FDA, OPDA, 4MPD, IPrDA, and ODA were dried overnight before use in a vacuum oven at 150 °C. Jeffamine was dried at 70 °C in a vacuum oven overnight prior to use.

### 2.2. Synthesis of Aromatic and Segmented (Co)polyimides

In this work two groups of polyimides were synthesized. The aromatic polyimides (**PI-1**–**PI-4**) were synthesized by a polycondensation reaction of the dianhydride (OPDA or 6FDA) with an equimolar amount of aromatic diamine (IPrDA, 4MPD, or ODA). The monomers were dissolved in NMP (20% total monomer concentration), and the reaction mixture was stirred for 18 h at room temperature under an argon atmosphere to make a viscous poly(amic acid) (PAA) solution. Then, toluene, as a low boiling azeotropic agent, was added to the PAA solution, and azeotropic distillation was performed at 170–185 °C for 5 h. After that, the polymer solution was cooled to room temperature, precipitated in methanol, and a polymer was collected by filtration and purified by Soxhlet extraction for a few days to remove any reaction solvents. Then, the polymer was dried in a vacuum oven at 100 °C for several hours.

The segmented copolyimides (**coPI-1**–**coPI-4**) were synthesized in the same way except that both diamines, aromatic diamine (x mmol) and Jeffamine (y mmol) with a molar ratio of 3:1, were incorporated separately into a reaction mixture. An aromatic diamine was the first to be dissolved in anhydrous NMP, then a stoichiometric amount of dianhydride (x + y mmol) was added in one portion, and the reaction mixture was stirred at room temperature for about 60 min under an argon atmosphere to obtain a clear solution. After that, Jeffamine (y mmol) was slowly added to the flask, and the reaction was carried out at room temperature overnight. Next, the copoly(amic acid) (coPAA) solution was thermally imidized at 160–185 °C for 5 h in the presence of toluene as azeotropic agent. The obtained products are labeled as follows: **PI-1** (OPDA, IPrDA), **PI-2** (OPDA, 4MPD), **PI-3** (OPDA, ODA), **PI-4** (6FDA, 4MPD), and the same description applies to the respective **coPI**s, e.g., **coPI-1** (OPDA, IPrDA, Jeffamine).

### 2.3. Membrane Formation

Polyimide-based membranes were prepared from the solution of a polymer (0.3 g of **PI**) in 5 mL of DMF (or in NMP in the case of **PI-3**), filtered through a 0.5 μm filter and cast onto a glass plate. The membranes were soft dried at 50 °C for 3 days in air, and then heated for another 3 days in a vacuum oven at a temperature gradually rising from 80 to 150 °C. Finally, the clear polymeric films were heated at 300 °C in an argon atmosphere for 2 h. Copolyimide-based membranes were obtained by casting **coPI** solution, diluted with DMF and filtered through a 0.5 μm filter, onto a Teflon plate mounted in a metal mould. The same drying procedure applied for the **PI**-based membranes was also used for the **coPI**-based ones except for the final drying stage, which consisted of heating in a vacuum oven at 200 °C for 16 h. The thickness of the membranes, calculated as an average of several thickness measurements, was in the range of 45–90 μm.

### 2.4. Measurements

Fourier transform infrared (FTIR) spectra were acquired on FTS 40 A Fourier transform infrared spectrometer (Bio-Rad, Digilab Division, Cambridge, MA, USA) between 4000–400 cm^−1^ at a resolution of 2 cm^−1^ and for 32 accumulated scans. Samples were analyzed as KBr pressed pellets or films. Proton nuclear magnetic resonance (^1^H NMR) spectra were recorded on an Avance II Ultra Shield Plus Bruker MT 600 MHz spectrometer using chloroform (CDCl_3_) as a solvent and TMS as the internal standard.

The X-ray diffraction data (WAXD) of the film samples were recorded using CuKα radiation (wavelength λ = 1.54051 Å) on a wide-angle HZG-4 diffractometer (Carl Zeiss, Jena, Germany) working in the typical Bragg geometry. The X-ray diffraction angle *Θ*, at which the maximum of a broad peak appeared on the WAXD profile, and the following Bragg’s expression:

(1) λ=2d⋅sinΘ

were used for calculation of an average interchain distance called *d*-spacing. The surface of the membranes was studied using the atomic force microscopy (AFM) on MultiMode 3d (di-Veeco, CA) working in the tipping-mode.

Differential scanning calorimetry (DSC) measurements were performed with a TA-DSC 2010 apparatus (TA Instruments, Newcastle, DE, USA) under nitrogen using a heating/cooling rate of 20 °C min^−1^. The glass transition temperature value (*T*_g_) was taken as the midpoint of the heat capacity step change observed at the second run. Thermogravimetric analysis (TGA) was carried out with a Thermal Analysis Q-1500 instrument (Hungarian). The 50 mg samples cut from the films were heated from 20 to 820 °C with a heating rate of 10 °C min^−1^, under constant flow of 7 L/h of nitrogen.

The gas permeation properties were investigated using a constant volume/variable pressure method in accordance with the procedure described earlier [30]. Degassing of the membrane was carried out by applying a vacuum in both feed and downstream sides for over 10 h. The measurements were carried out at 30 °C under 6 bar pressure. The gas permeability (*P*) of the pure gases O_2_, N_2_, He, and CO_2_ was obtained from the following formula:

(2) $$P=10^{-10}\frac{V_{d}\cdot l}{A\cdot T\cdot R\cdot p_{2}}\left[{\left(\frac{dp_{1}}{dt}\right)}_{ss}-{\left(\frac{dp_{1}}{dt}\right)}_{leak}\right]$$

where *P* is the permeability [Barrer]; *V_d_* is the downstream volume [cm^3^]; *l* is the membrane thickness [cm]; *A* is the effective membrane area [cm^2^]; *T* is the absolute temperature [K], R is the gas constant [cm Hg cm^3^ cm^−3^ (STP) K^−1^]; *p*_2_ is the feed absolute pressure [cm Hg]; $\frac{dp_{1}}{dt}$ is the rate of permeate absolute pressure rise for steady-state (“*ss*” index) and leak conditions state (“*leak*” index).

The ideal selectivity was calculated from a single gas permeation experiment as:

(3) $$\alpha_{\left(A/B\right)}=\frac{P_{A}}{P_{B}}$$

## 3. Results and Discussion

### 3.1. Characterization of (Co)polyimides

Chemical structures of (co)polyimides studied in this work are presented in Scheme 1.

**Figure 1:**
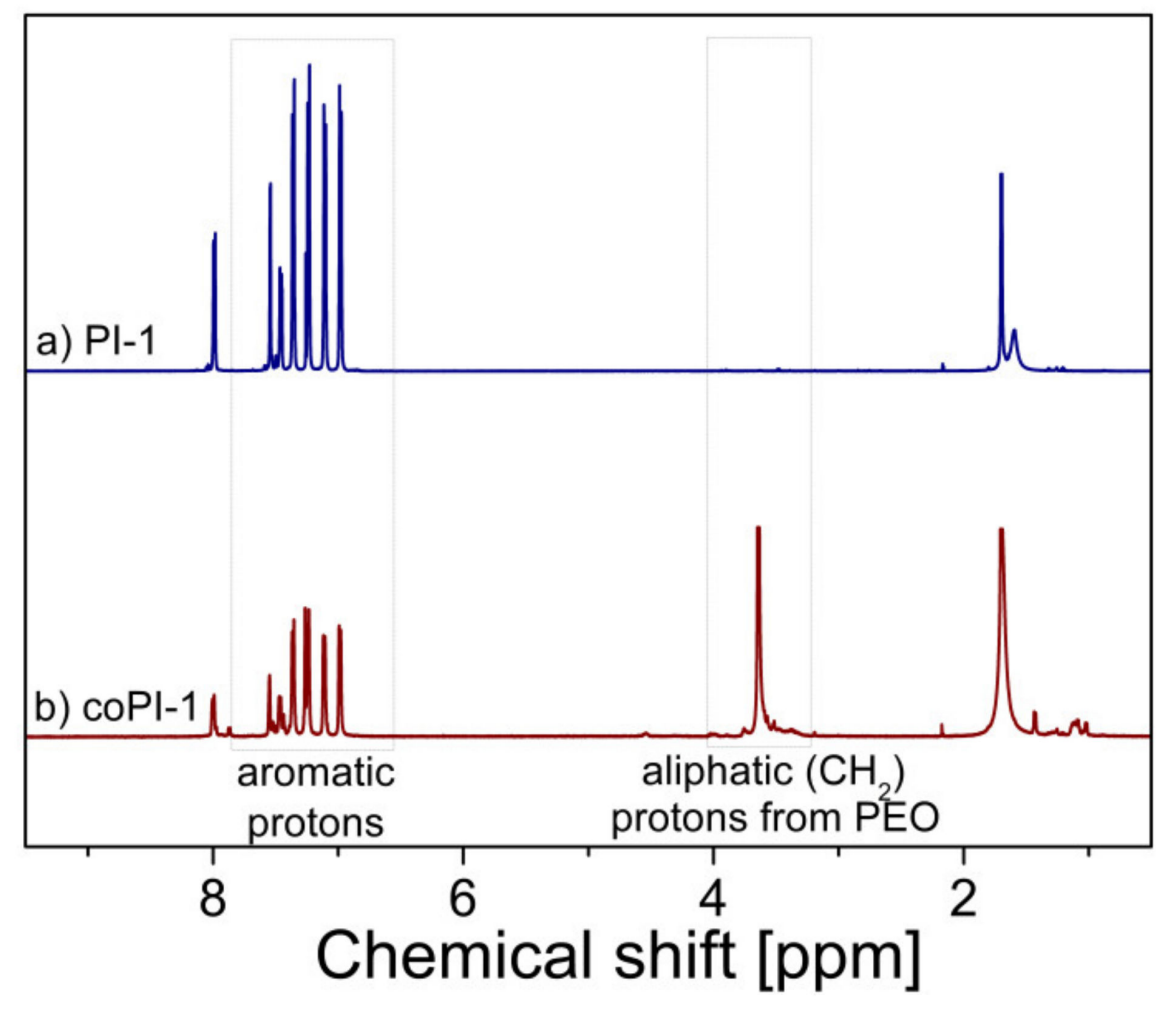
Proton nuclear magnetic resonance (^1^H NMR) spectra of (co)polyimides: (**a**) **PI-1**, (**b**) **coPI-1**.

These structures were confirmed by ^1^H NMR and FTIR spectroscopy. The ^1^H NMR spectra of **PI-1** and **coPI-1** are shown in Figure 1 as an example.

The positions of the signals on these spectra correspond to the protons coming from the expected chemical moieties in the synthesized polymers. For both **PI-1** and **coPI-1**, the aromatic proton resonances are in the same range of 7–8 ppm, while the methyl protons in the aromatic diamine unit (IPrDA) are seen as a singlet at about 1.70 ppm. For **coPI-1**, the presence of additional proton resonance at 3.64 ppm (Figure 1b) assigned to methylene groups from PEO indicates that copolymerization was successful. For all of the synthesized PIs and coPIs, the spectra are without any resonances above 10.5 ppm, which correspond to the carboxylic acid proton. This is an indication of a complete conversion of poly(amic acid) to polyimide.

**Figure 2:**
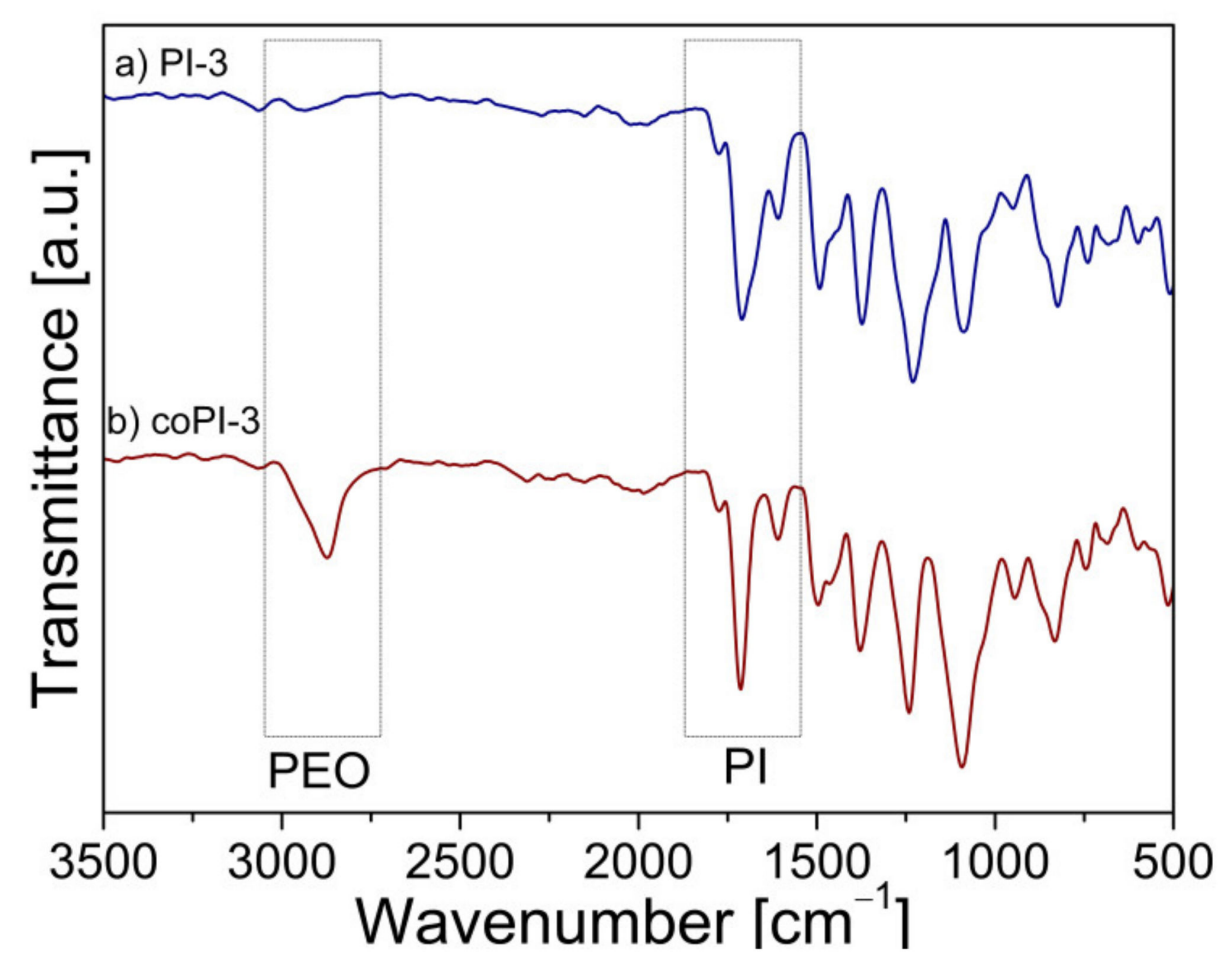
Fourier transform infrared (FTIR) spectra of (co)polyimides: (**a**) **PI-3**, (**b**) **coPI-3**.

The fully imidized structure of PIs and coPIs was also confirmed by the lack of absorption bands at 1650 cm^−1^ (amide group), and at 3350 cm^−1^ (carboxyl group) in the ATR-FTIR spectra. Instead, as shown in the representative spectra of **PI-3** and **coPI-3** given in Figure 2, all (co)polyimides exhibited characteristic imide ring absorbances: at 1774–1778 cm^−1^ (symmetric C=O stretch), 1712–1723 cm^−1^ (asymmetric C=O stretch), 1351–1377 cm^−1^ (C-N-C stretch), and around 745 cm^−1^ (C-N-C ring deformation). For coPIs, the presence of the aliphatic polyether segments was evident by a strong absorption band around 2870 cm^−1^ (Figure 2b). The synthesized polymers exhibited an excellent solubility in polar aprotic media such as NMP, DMF (except for **PI-3**), DMSO and even in chloroform, and tetrahydrofuran. The improved solubility of the obtained polymers compared with that of conventional polyimides can be explained by the presence of flexible ether bridges in the polymer chains together with groups that introduce a disruption in the chain packing.

### 3.2. Thermal Properties

**Figure 3:**
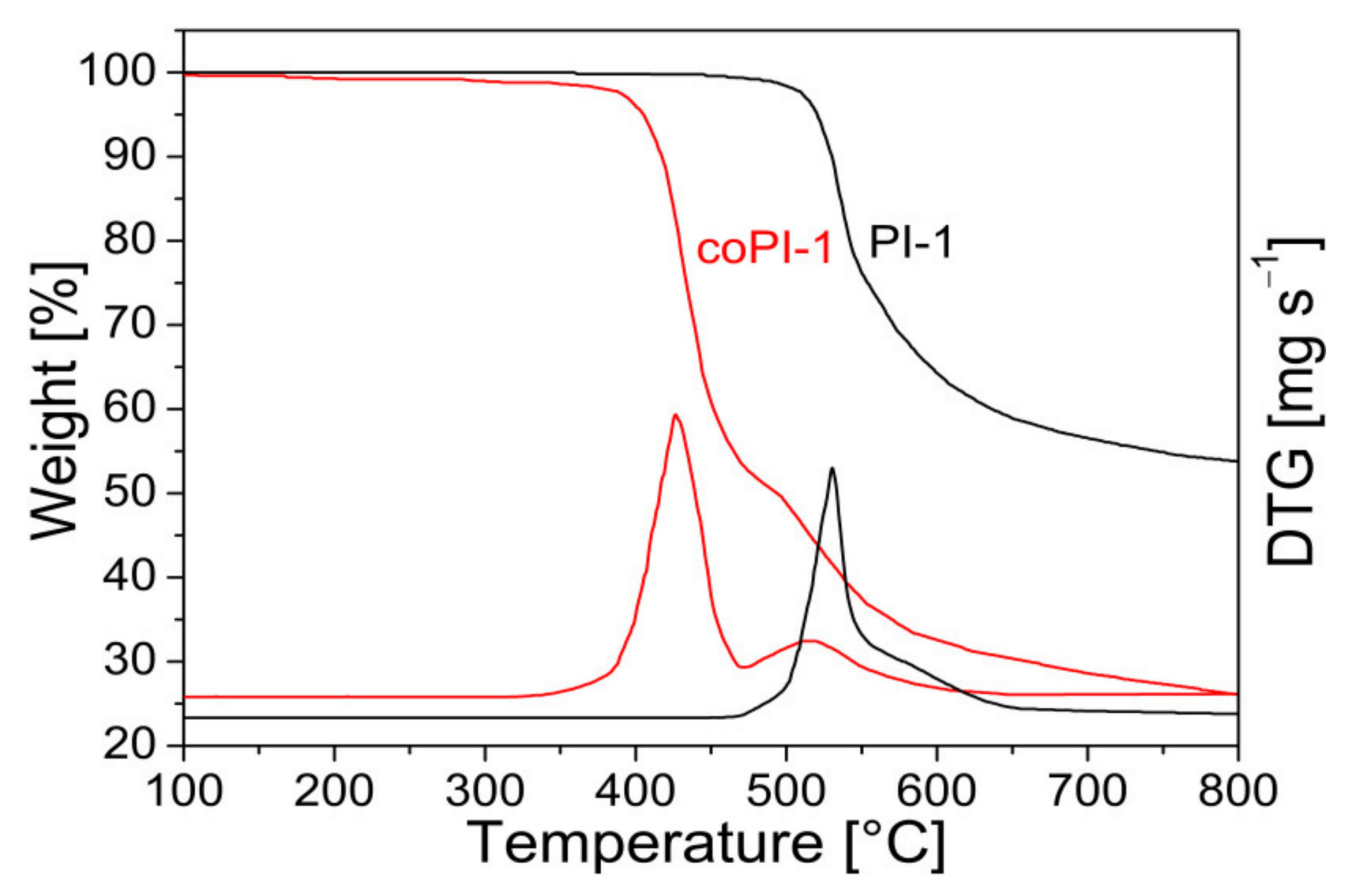
Thermogravimetric analysis (TGA) of **PI-1** and **coPI-1**.

Thermogravimetric analysis was performed to evaluate the thermal stability of the synthesized (co)polyimides. Figure 3 shows representative TGA thermograms for PI and the corresponding coPI, whereas the results of the TGA analysis for all of the samples are listed in Table 1.

As demonstrated by the profiles in Figure 3, PI features a single thermal degradation stage in contrast to two distinct weight loss stages observed for coPI. Since this second weight loss in the TGA profile of coPI occurs in the similar temperature region as for PI (above 500 °C), it can be attributed to the thermal decomposition of the aromatic polyimide segments. Consequently, the first weight loss in the TGA thermogram of coPI corresponds to the degradation of the PEO soft segments. The temperature of maximum weight loss rate for this step (*T*_1_) is in the range of 400–420 °C for all the copolyimides. The comparison of weight loss during this stage with the PEO content calculated based on the composition of the monomers shows that there is a reasonably good agreement between the experimental and the calculated amount of the soft segments in coPIs (Table 1). Similarly, the differences in the char residue values between PI and the respective coPI are in accordance with the corresponding differences in the aromatic segment content in both kinds of materials.

**Figure 4:**
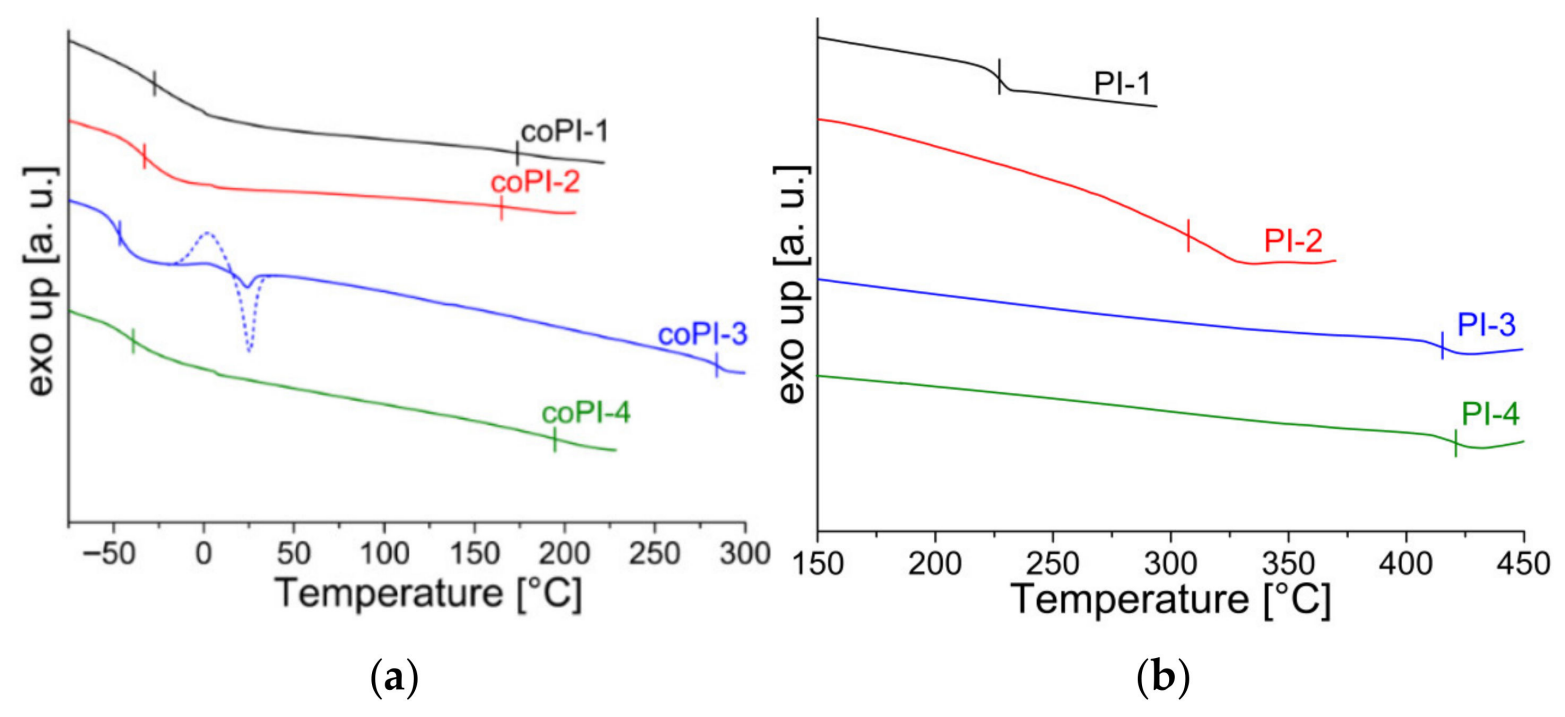
Differential scanning calorimetry (DSC) thermograms of the synthesized (co)polyimides; (**a**) segmented coPIs, (**b**) aromatic PIs.

Figure 4a demonstrates the DSC curves for the studied copolymers, whereas Figure 4b shows the scans for the respective fully aromatic polyimides. The copolymers have two glass transition temperatures, one in the sub-ambient temperature region, and the second in the high temperature range, where the glass transition of the aromatic polyimides is expected. The presence of two glass transitions indicates that the copolymers have a microphase separated morphology. This morphology can be described as consisting of polyimide-based hard domains and soft domains, where the polyether segments are predominating. The glass transition temperature (*T_g_*) values of both phases (*T*_g_1 and *T*_g_2) are shown in Table 1. The higher *T_g_* determined for the PEO phase of the studied coPIs (*T*_g_1) compared to that of Jeffamine 2003 (−58 °C) implies that some of the PI hard segments have been mixed into the PEO phase imposing mobility restrictions on it. As demonstrated by the *T*_g_1 values, the extent of segment thermodynamic incompatibility responsible for phase separation depends on the chemical structure of the coPI hard segments. It seems to be higher for polyimide segments with stronger intermolecular forces, as is the case with **coPI-3**, which, according to its lowest *T*_g_1, shows the highest degree of phase separation. Moreover, in this copolymer, the PEO segments possess sufficient freedom of movement to allow crystallization, as suggested by a small endothermic peak observed at 25 °C. Both the crystallization and melting of the PEO crystals in this copolymer can clearly be seen in the consecutive DSC scan after pre-heating to 250 °C (dotted line in Figure 4a). Thus, from the obtained result it can be deduced that PEO segments in **coPI-3** form a soft phase of a relatively high purity. It should also be noted that when measuring permeation properties at 30 °C, this soft phase is fully amorphous.

For all of the studied coPIs, the *T_g_* values of the hard segment domains (*T*_g_2) are lower than the values of the respective fully aromatic polyimides. This can be due to the lower molar mass of the coPI hard segments compared to that of the respective PI homopolymer, as well as to the possible presence of residual solvent molecules resulting from a lower drying temperature applied to coPI films, or to the inclusion of some polyether segments in the hard domains. The comparison of the *T_g_* values of aromatic polyimides shows that 6FDA-based polyimide (**PI-4**) has a higher *T_g_* than an OPDA-based one (**PI-2**) due to the restricted torsional motion of phenyl rings around a C(CF_3_)_2_ linkage. On the other hand, the significantly decreased chain stiffness, as shown by the lowest *T_g_* value, can be observed for **PI-1** with the highest diphenylether linkage concentration.

### 3.3. Wide-Angle-X-ray Diffrection Patterns

**Figure 5:**
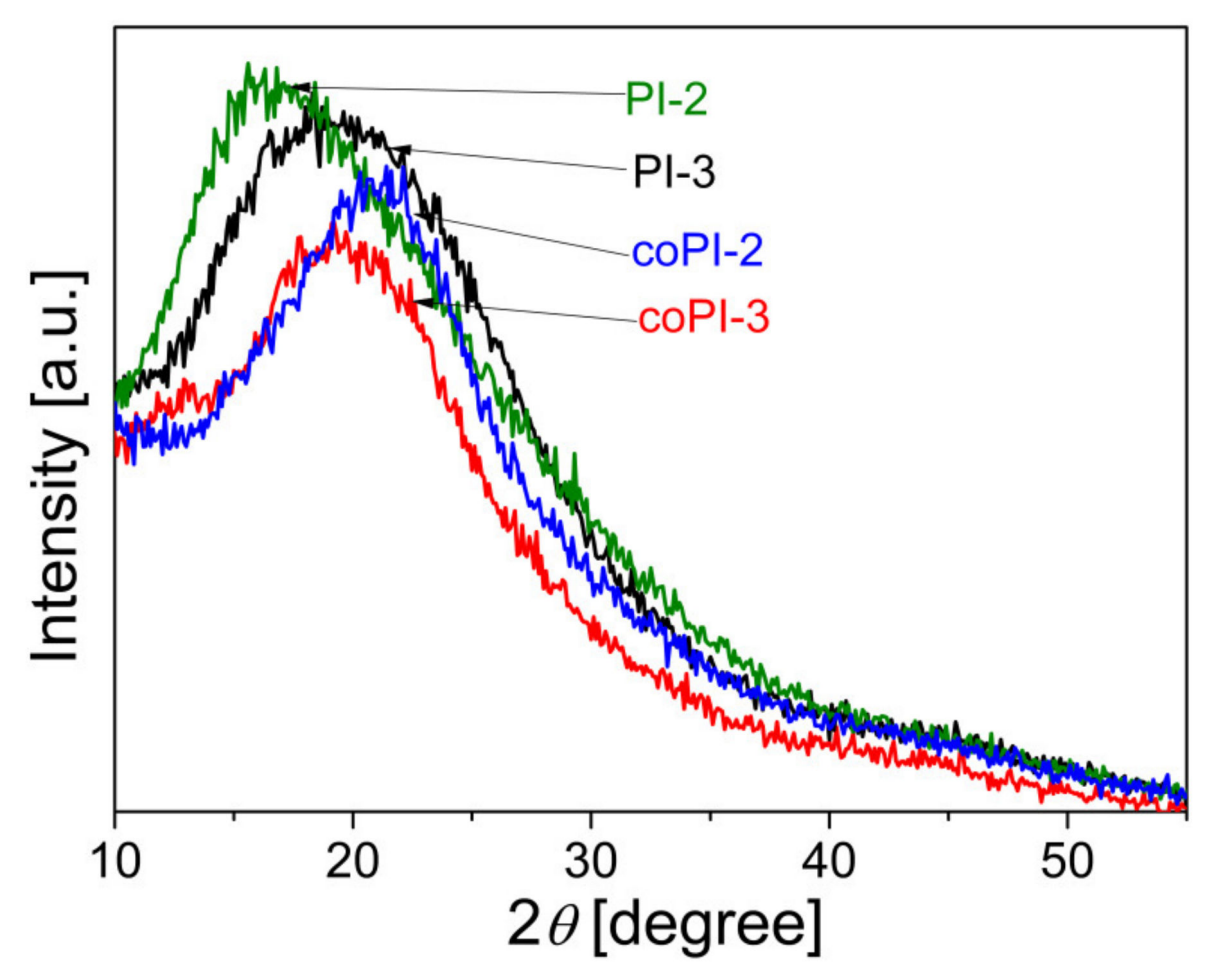
Wide-angle X-ray diffraction (WAXD) patterns of aromatic **PI-2** and **PI-3**, and segmented **coPI-2** and **coPI-3**.

Wide-angle X-ray diffraction measurements for the PI and coPI membranes were performed to investigate their morphology and to determine the impact of PEO segments on the chain packing in coPIs. Representative WAXD patterns recorded at room temperature are shown in Figure 5.

All of the studied membranes exhibit a broad halo lacking in any crystalline peaks, which indicates their fully amorphous nature. The *d*-spacing values calculated from the position of the diffraction maximum are summarized in Table 1. As can be seen from these data, the *d*-spacing of each copolyimide is smaller than that of its fully aromatic counterpart. This effect is more distinct for PI with reduced intra- and inter-chain interactions and looser chain packing, such as in the case of **PI-2** and its segmented analogue. Thus, these results suggest that PEO segments, by imparting some flexibility to the polymer chains, allow them to adopt a more favorable conformation, which results in tighter polymer chain packing. The differences in chain packing can also be seen among the aromatic PIs. 6FDA-based PIs with the bulky C(CF_3_)_2_ group in their structure (**PI-4** and **coPI-4**) have larger *d*-spacing values than the other PIs based on OPDA dianhydride. On the other hand, among the OPDA-based aromatic PIs, **PI-2** has the highest *d*-spacing due to four CH_3_ groups connected to the phenyl ring in the diamine moiety, which restrict efficient packing by steric hindrance, while **PI-3** shows the opposite effect of the lowest *d*-spacing value due to the presence of flexible diphenylether linkages.

### 3.4. Surface Morphology Characterization by AFM

**Figure 6:**
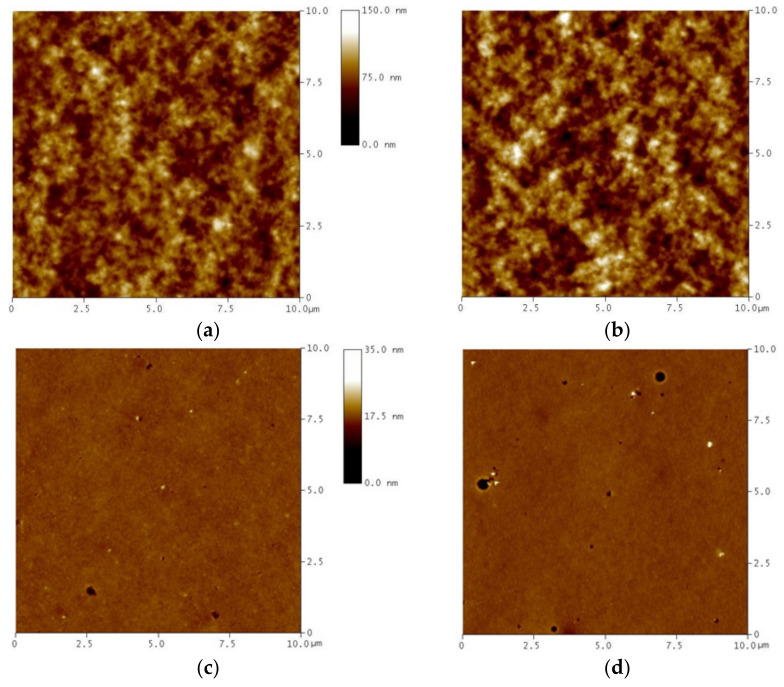
Atomic force microscopy (AFM ) height images of segmented coPIs (the top row) and aromatic PIs (the bottom row), (**a**) **coPI-1**, (**b**) **coPI-3**, (**c**) **PI-1**, (**d**) **PI-3.**

The 2D AFM images of surfaces of the selected segmented and aromatic polyimides are shown in Figure 6. As can be seen from this figure, the morphologies of both families of polymers present distinctly different features, while within the given family they are similar in appearance. The surfaces of the segmented polyimides (Figure 6a,b) seem to be much coarser than those of the aromatic polyimides. They show numerous bright and dark spots, which correspond to the highest and lowest regions of the surface. The areas varying in height are irregular in shape and size, highly interconnected and relatively uniformly distributed throughout the sample. In contrast, the aromatic polyimides present very smooth surfaces without any detectable features except for a few small holes, which seem to be a result of the solvent evaporation process (Figure 6c,d).

To characterize the variations in surface roughness, the RMS parameter, defined as the root mean square average of height deviations from the mean data plane, was determined. Since this parameter is the scale dependent, all roughness measurements presented here were calculated for the images of the same surface area of 10 µm × 10 µm. From the obtained RMS data, which are 12.590 nm and 14.941 nm for **coPI-1** and **coPI-3**, respectively, and equal to 1.525 nm and 1.878 nm for **PI-1** and **PI-3**, respectively, it is evident that the surface of segmented coPIs is about one order of magnitude coarser than that of aromatic PIs. The smoother surface of aromatic PIs can be explained by the presence of imide groups along their chains, which have the effect of increasing the intermolecular forces and keeping the main chains close together. On the other hand, the AFM images of segmented coPIs provide a visual confirmation of two phase morphology of these samples, where flexible and rigid chain segments are capable of forming different regions.

### 3.5. Gas Transport Properties

The permeability coefficients of the aromatic and segmented (co)polyimides to CO_2_, O_2_, N_2_, and He, and the ideal selectivity values are reported in Table 2.

The observed permeabilities of the aromatic PIs, except for **PI-4**, follow the order of He > CO_2_ > O_2_ > N_2_, which is consistent with the order of increasing gas kinetic diameters. This behavior is typical for many polyimides and other glassy polymers [32,33], where diffusivity controls permeability. However, for the segmented coPIs, as well as for **PI-4**, the sequence of the gases takes a different form of CO_2_ > He > O_2_ > N_2_. The observed higher permeability of CO_2_ compared to that of He, despite its larger size, indicates that CO_2_ permeability in those polymers is largely determined by the solubility factor. The increase in solubility contribution to CO_2_ permeability in coPIs is due to favorable interactions between CO_2_ molecules and polar PEO segments. This effect was observed by us earlier for random copolymers (EVA) with polar vinyl acetate groups [34], and also reported by other authors with respect to amorphous PEO [35] and some PEO-based segmented copolymers [16,19]. However, for glassy **PI-4**, the origin of CO_2_ solubility increase is not the same. In this case, it is due to an exceptionally high fractional free volume of **PI-4**, resulting from the bulky groups present in both dianhydride (CF_3_ linkage) and diamine (CH_3_ on the phenyl ring) moieties, which enhances the solubility of easily condensable gases. Similar results of a higher CO_2_ permeability than that of He have also been reported in the literature for other high free volume polyimides, such as 6FDA-DAM [36].

As can be seen from Table 2, the favorable increase of CO_2_ permeability in coPIs, caused by its interactions with the PEO segments, results in a very high CO_2_/N_2_ selectivity with values reaching up to 67 that extends those reported for poly(ethylene oxide) [35]. This is in stark contrast to CO_2_/N_2_ selectivity of the aromatic PIs, which is in the range of 13 to 29 showing a typical trade-off between permeability and selectivity. The notion that massive gain in the CO_2_/N_2_ selectivity of coPIs is mainly due to solubility enhancing interactions with CO_2_ is further supported by their substantially lower selectivities for the O_2_/N_2_ and He/N_2_ gas pairs when compared to the respective aromatic PIs (except for **coPI-4** and its **PI-4** analogue, which will be discussed later). Based on these data, it appears that coPIs under this investigation, which contain 49–55 wt.% of PEO, display the behavior of rubbery polymers with a limited ability to discriminate molecules in terms of their size. On the other hand, while the CO_2_/N_2_ selectivity of coPIs seems to be controlled by the PEO-based soft domains, without too much of a variation within a series regardless of the molecular structure of the polyimide hard segments, the permeability of those materials turns out to be related to the hard segment structure. The most permeable **coPI-3** membrane exhibits a permeability for CO_2_ that is 8 times higher than that of the least permeable **coPI-2**. However, the observed permeability differences among coPIs are much lower compared to those found for the aromatic PIs, where in the extreme case, CO_2_ permeability is around 920 times higher for **PI-4** than for **PI-3**. Moreover, the obtained following order of gas permeability through coPIs, **coPI-3** > **coPI-4** > **coPI-1** > **coPI-2**, is different from that found for the series of aromatic PIs, which is as follows: **PI-4** > **PI-2** > **PI-1** > **PI-3**. A dramatic drop in permeability has been observed when PEO sequences are incorporated into **PI-4** and **PI-2** polyimides containing 4MPD moieties in their chains. As great as 87 fold and 19 fold permeability decreases for O_2_ and CO_2_ transport, respectively, is recorded for **coPI-4** compared to aromatic **PI-4**. For the less permeable **PI-2**, the respective declines in **coPI-2** permeability are correspondingly lower, 21 and 5 fold for O_2_ and CO_2_, respectively. The decrease of permeability for these materials may be explained by the reduced diffusivity through the copolymer hard segment domains as a result of a partial filling of the free voids in the hard domains by PEO segments or by residual solvent molecules. This explanation seems to be supported by the assessment based on the *Tg* values of the copolymers hard domains. Both materials show a considerable decrease of glass transition temperature values (*T_g_*2) (Table 1), which can be assigned to the plasticization of their hard domains. This interpretation also coincides with the slightly improved selectivity of **coPI-4** towards the He/N_2_ gas pair. On the other hand, the significant decrease of the permeability coefficients for **coPI-4** and **coPI-2**, which leads these materials to rank behind **coPI-3**, cannot be explained by the reduced hard segment diffusivity alone, especially that permeability of the aromatic **PI-3** is remarkably lower than that of the other PIs, in particular that of **PI-4**. As the copolymers studied in this work possess the same soft/hard composition of close to 50/50, the observed drop in **coPI-4** and **coPI-2** permeability has to be associated with an increased density of their soft domains. This may result from imperfect phase separation and the presence of some hard segments in the soft domains, which gives rise to the reduced flexibility of PEO segments and decreased diffusivity. While all gases experience permeability decline, CO_2_ transport is less affected because of the strong interactions between CO_2_ and PEO, which increase solubility and partially compensate for this loss in diffusivity. The differences in the soft domain *T_g_* values listed in Table 1 (*T_g_*1) seem to correlate with the observed permeability variations. As shown, the highest gas permeability of **coPI-3** is in accordance with the lowest glass transition temperature of its soft domains, which corresponds to the highest degree of phase separation and hard segment free soft domains.

**Figure 7:**
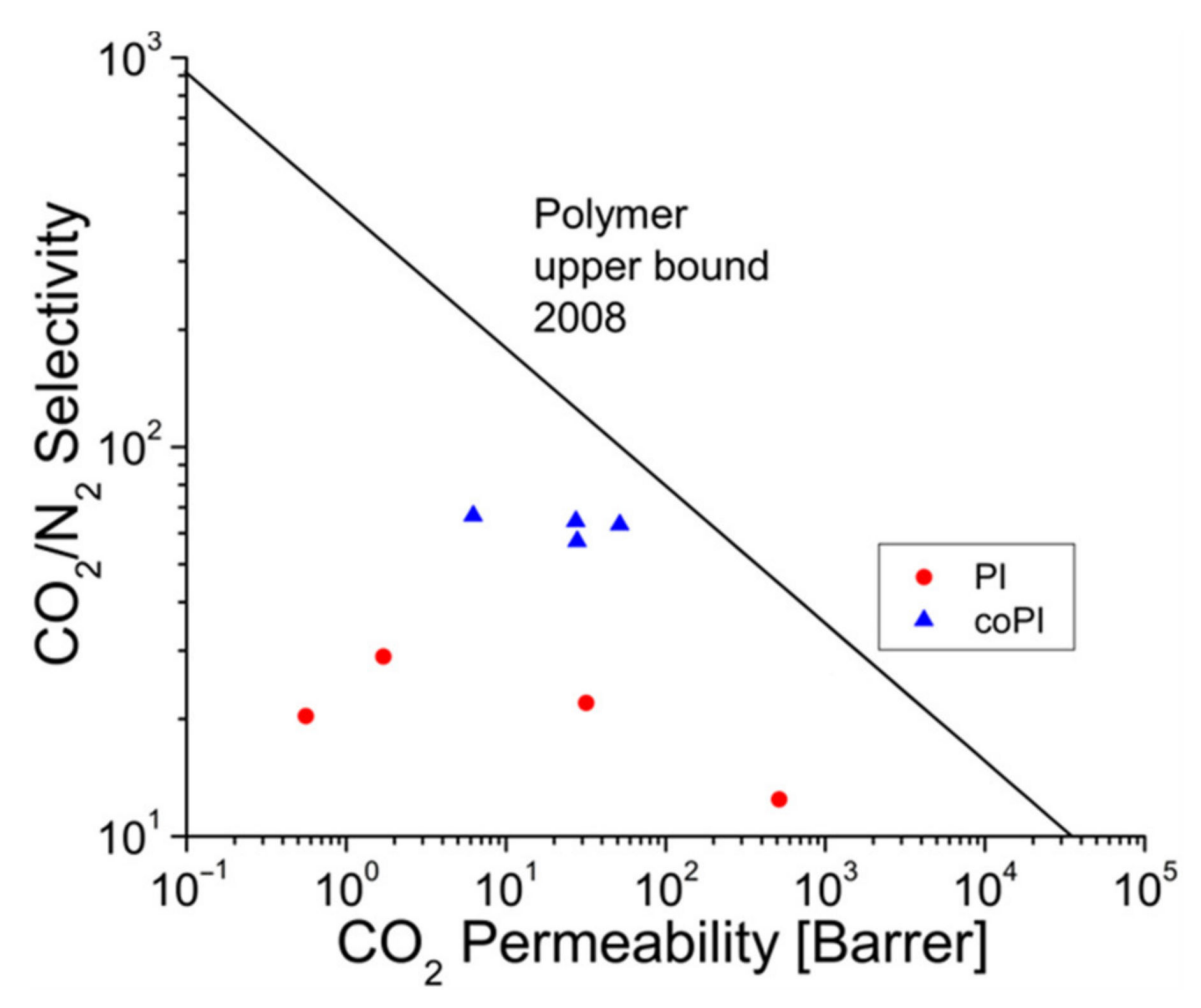
Roberson’s plot for the CO_2_/N_2_ gas pair.

In Figure 7, the values of permeability and selectivity for the CO_2_/N_2_ gas pair are presented for both families of the membrane materials in a form of the Roberson’s diagram. Comparing the membrane separation efficiency with the Roberson’s upper bound allows for convenient assessment of membrane performance. As depicted in Figure 7, the copolymers in this study exhibit very high selectivity towards the CO_2_/N_2_ gas pair. With the modification of a hard segment chemical structure, the permeability of the respective copolymers increases and the coPI samples shift towards the upper bound. Since this shift occurs without sacrificing selectivity, it clearly overcomes the permeability–selectivity trade off. In contrast, all of the aromatic polyimides are far away from the upper bound, which indicates their relatively low performance.

**Figure 8:**
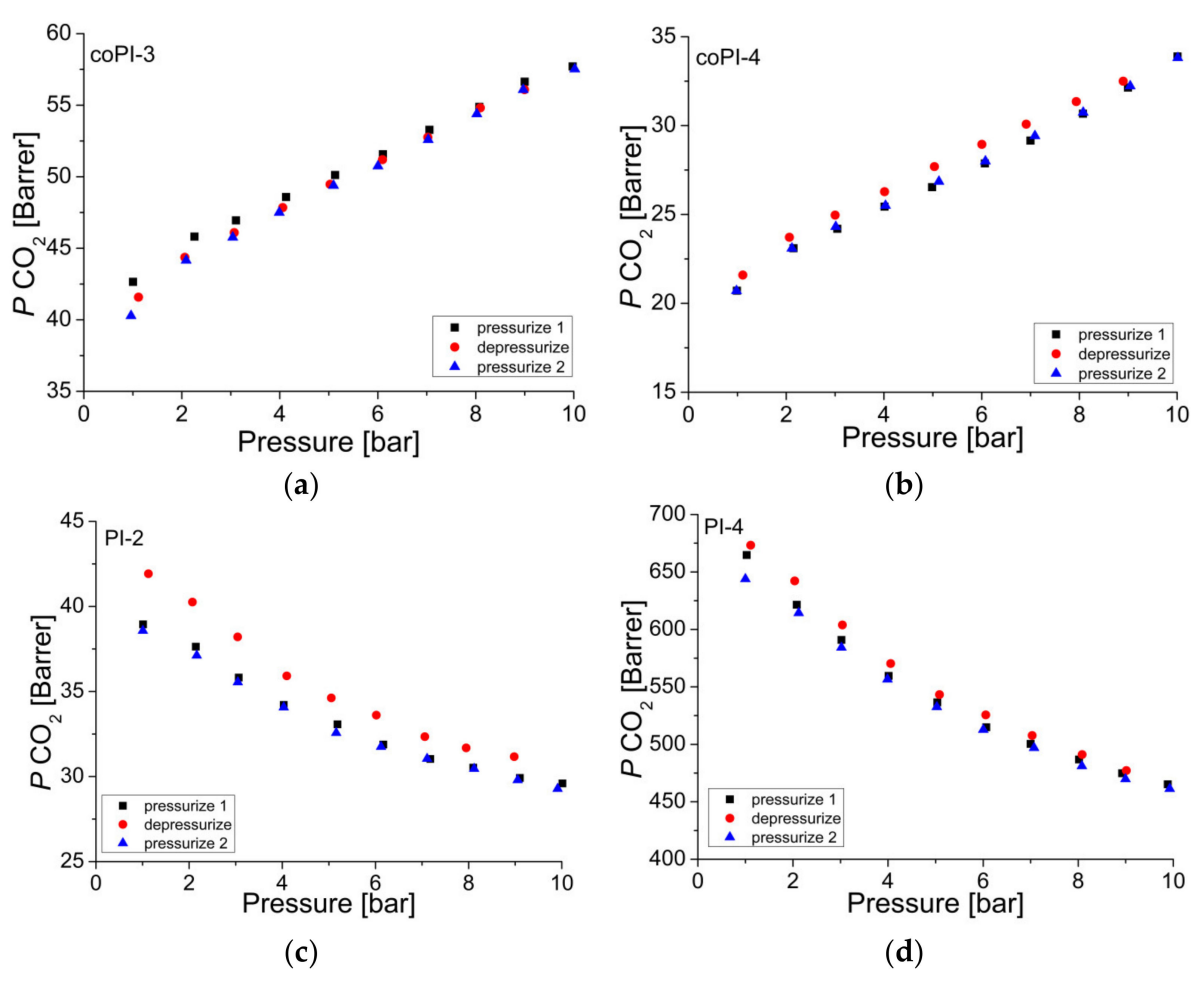
Pure CO_2_ permeability of copolyimide: (**a**) **coPI-3**, (**b**) **coPI-4**, and aromatic polyimide: (**c**) **PI-2**, (**d**) **PI-4** membranes as a function of feed pressure at 30 °C.

The effect of the CO_2_ transmembrane pressure on permeability of the membranes based on segmented polyimides was studied and compared with the results obtained for the fully aromatic polyimides. As can be seen in Figure 8, for the coPI-based membranes, CO_2_ permeability increases as the pressure is progressively increased, whereas the opposite trend of decreased permeability is observed for the membranes based on aromatic polyimides. The permeation behavior of CO_2_ in coPI is typical for rubbery polymers, and originates from the enhanced CO_2_ sorption at elevated pressures leading to plasticization of the polymer matrix [37,38]. The recorded permeability changes during the consecutive pressurization, depressurization, and the second pressurization cycles show no hysteresis in the case of **coPI-3** (Figure 8a) indicating a quick response of the flexible polymer matrix to the pressure variations. However, a slight delay in the de-swelling of the polymer matrix during the depressurization cycle can be noticed for **coPI-4** with a higher degree of phase mixing, and, thus, less mobile soft segments (Figure 8b). In contrast, the investigated aromatic polyimides show no plasticization response for the studied feed pressures, up to 10 bar. The observed permeability decrease (Figure 8c,d) with increased pressure is characteristic of dual-mode transport, and is due to the gradual saturation of the Langmuir sites in glassy polymers [39]. The overall permeability reduction is 24%, and 30% for **PI-2** and **PI-4**, respectively. The larger drop in permeability for **PI-4** than for **PI-2** is due to its higher free volume. As appears from the *FFV* values determined for both polymers, which are 0.18 and 0.14 for **PI-4** and **PI-2**, respectively, *FFV* of **PI-4** is higher by 22%. These values were calculated based on the measured densities (1.333 g/cm^3^ and 1.26 g/cm^3^for **PI-4** and **PI-2**, respectively) and the Bondi’s group contribution method [40] as described elsewhere [31]. Upon completion of the depressurization cycle, the depressurization curve lying above the pressurization one can be seen, which is a common effect for glassy polymers showing time dependent relaxations. The results of the second pressurization step, performed after the evacuation of the membranes in a vacuum for 16 h, are basically in line with those from the first one. The observed plasticization resistance of the aromatic PIs to the applied pressures is consistent with the results of other authors reporting that CO_2_ induced plasticization of the 6FDA-4MPD polyimide starts at 10 bar or 30 bar depending on the membrane thermal history [41]. It is also worth mentioning that thermal treatment of the membranes based on aromatic polyimides adopted in this work (300 °C) reduced CO_2_ permeability of **PI-4** by 18% compared to the permeability reported by us previously for the **PI-4** membrane treated at 150 °C [42]. This behavior can be related to the reduction of the excess free volume and a tighter packing of polymer segments in the membrane caused by lattice contraction during high temperature annealing.

## 4. Conclusions

A series of the new PEO-based rubbery segmented copolyimides varying in hard segment chemical structure and an analogues series of fully aromatic glassy polyimides were synthesized and the ability of these polymers to separate carbon dioxide from flue gas mixture was studied at 30 °C and at a pressure of up to 10 bar using pure gases. The structural variations were designed by carefully selecting dianhydride-aromatic diamine combinations, which resulted in fully aromatic polyimides differing by as much as 920 times in CO_2_ permeability. The application of the same monomer combinations to create hard segments of the PEO-based copolyimides produced membranes with distinctly different gas transport properties. The difference in CO_2_ permeability between copolymer membranes turned out to be much smaller, only 8 fold, whereas the selectivity appeared to be basically independent (or very slightly dependent) on the hard segment chemical constitution. The OPDA-ODA-based copolymer, the fully aromatic analogue of which ranked as the least permeable in the polyimide series, proved to be the most permeable copolymer. On the other hand, the 6FDA-4MPD monomer combination, which gives one of the most permeable aromatic polyimides, when used to form the PEO-based segmented copolymer resulted in a material that is half as permeable as the most permeable OPDA-ODA-based copolymer. The decreased permeability of the copolymer compared to its aromatic counterpart was also observed in the case of the second 4MPD-based polymer studied here. This was attributed to the reduced diffusivity through both the 4MPD-based hard domains, as a result of a partial filling of their free voids by PEO segments, and the soft domains due to their increased density resulting from the less pronounced phase separation between the PEO and 4MPD-based phases. The impact of the hard segment structure on transport properties was found to be consistent with the trends of both the hard and soft segment *T*_g_ variations determined from the DSC studies. Thus, the results of this work show that the chemical composition of the hard segment plays an important role in obtaining high performance membrane materials. Although the incorporation of a highly permeable polyimide moiety (6FDA-4MPD) into a PEO-based segmented copolymer failed to combine the superior properties of both glassy and rubbery components, the selected aromatic monomers allowed membrane materials with a very high CO_2_/N_2_ selectivity (above 60) and diverse permeability to be obtained. Among them, the OPDA-ODA-PEO copolymer with a CO_2_ permeability of 52 Barrer and CO_2_/N_2_ selectivity of 63 ranks among the best performing materials of this type reported in the literature.

## Figures and Tables

**Table 1:** Thermal properties and *d*-spacing values of the aromatic and segmented (co)polyimides.

Code	Structure	TGA					DSC		WAXD
		PEO Content[wt.%]		T_max_^a^ [°C]		Char Yield ^b^ [%]	*T_g_* [°C]		*d*-Spacing [Å]
		calc.	TGA	*T*_1_	*T*_2_		*T*_g_1	*T*_g_2	
**PI-1**	ODPA-IPrDA				550	54		227	5.09
**PI-2**	ODPA-4MPD				520	58		307	5.33
**PI-3**	ODPA-ODA				600	59		415	4.71
**PI-4 ^c^**	6FDA-4MPD				546	55		421	6.1
**coPI-1**	ODPA-IPrDA-PEO 3:1	44.6	49	420	515	27	−28	174	4.67
**coPI-2**	ODPA-4MPD-PEO 3:1	55	49	400	520	31	−33	165	4.28
**coPI-3**	ODPA-ODA-PEO 3:1	51.6	54	400	570	30	−47	284	4.67
**coPI-4**	6FDA-4MPD-PEO 3:1	46.3	49	423	520		−39	194	5.48

^a^ Temperature of maximum decomposition rate; ^b^ Residual weight when heated at 800 °C in nitrogen; ^c^ [31] Jeffamine 2003, *T_g_*: −58 °C, *T_m_*: 44 °C; this work.

**Table 2:** Pure gas permeabilities and selectivities of the aromatic (PI) and segmented (coPI) (co)polyimides at 6 bar feed pressure and 30 °C.

Sample	Permeability [Barrer]				Selectivity		
	N_2_	O_2_	He	CO_2_	O_2_/N_2_	He/N_2_	CO_2_/N_2_
**PI-1**	0.059	0.446	9.05	1.71	7.56	153.4	29.0
**PI-2**	1.45	7.79	52.4	31.9	5.37	36.1	22.0
**PI-3**	0.028	0.199	2.89	0.56	7.11	103.2	20.0
**PI-4**	41.4	142.0	376	515	3.43	9.1	12.4
**coPI-1**	0.426	1.37	3.75	27.4	3.22	8.8	63.3
**coPI-2**	0.094	0.368	2.40	6.25	3.91	25.5	66.5
**coPI-3**	0.816	2.32	4.61	51.6	2.84	5.6	63.2
**coPI-4**	0.487	1.63	7.05	27.9	3.35	14.5	57.3
